# Nonlinear Dose–Response Association Between Exercise Intensity and Urinary Liver-Type Fatty Acid-Binding Protein in Young Men: A Piecewise Threshold Analysis

**DOI:** 10.3390/ijms27198539

**Published:** 2026-09-24

**Authors:** Hoa Do Thanh, Duan Duc Le, Tuan Nguyen Anh, Cuong Nguyen Thai, Ghi Nguyen Hai, Duong Le Xuan

**Affiliations:** 108 Military Central Hospital, No. 1 Tran Hung Dao Str., Hai Ba Trung Dist., Hanoi 100000, Vietnam; hoadoc13@gmail.com (H.D.T.); duanhocvienquany@gmail.com (D.D.L.); nguyentuanvmmu@gmail.com (T.N.A.); nguyenthaicuong108@gmail.com (C.N.T.)

**Keywords:** acute kidney injury, renal hemodynamics, proximal tubules, renal tubular stress, exertional ischemia, segmented regression

## Abstract

High-intensity physical exercise can induce temporary renal hemodynamic strain and subclinical tubular injury, yet the exact dose–response relationship remains poorly defined. This study investigated the relationship between exercise intensity and urinary liver-type fatty acid-binding protein (L-FABP) concentrations, a sensitive marker of proximal tubular stress, to identify an exploratory intensity threshold. We conducted an observational cohort study of 285 healthy young men undergoing a routine 20 min physical training session. Exercise intensity was quantified as metabolic equivalents of task (METs) using wearable accelerometers, and urinary L-FABP was measured pre-exercise and 30 min post-exercise. Post-exercise urinary L-FABP concentrations increased significantly from baseline (median, 1.89 vs. 0.90 µg/gCr; *p* < 0.001). Restricted cubic splines confirmed a nonlinear dose–response relationship (*p* for nonlinearity < 0.001) with an estimated breakpoint at 6.76 METs (95% CI, 5.67 to 7.12). Below 6.76 METs, exercise intensity was not significantly associated with post-exercise L-FABP (β = 0.068, *p* = 0.358), whereas above this threshold, L-FABP escalated rapidly (β = 0.491, *p* < 0.001). In conclusion, exercise intensity nonlinearly correlates with proximal renal tubular stress in young men. The 6.76 MET threshold represents an exploratory breakpoint marking an inflection zone beyond which exercise-associated subclinical renal tubular stress accelerates.

## 1. Introduction

Physical exercise significantly alters kidney function, particularly renal hemodynamics. During exercise, effective renal blood flow decreases in proportion to the exercise intensity, with strenuous activity potentially dropping renal blood flow to 25% of the resting value [1]. Exercise-induced reductions in renal blood flow, driven by increased sympathetic tone, elevated core temperature, muscle damage, and hypohydration, can cause renal ischemia and ATP depletion, thereby predisposing individuals to acute kidney injury [2]. Proximal tubular epithelial cells heavily depend on aerobic metabolism. They are particularly vulnerable to ischemia stress [3]. Liver-type fatty acid-binding protein (L-FABP) is a 14 kDa protein expressed in the proximal tubular epithelial cells [4], released into the urine in response to oxidative stress and ischemic or hypoxic injury [5]. Urinary L-FABP has been established as a clinical biomarker for detecting early subclinical renal tubular stress and injury across various clinical settings, including acute kidney injury, sepsis, and ischemic renal insults [6,7,8]. Unlike traditional clinical markers such as serum creatinine, which primarily reflect functional changes in glomerular filtration rate [9] and typically increase relatively late after acute kidney injury, urinary L-FABP serves as a sensitive biomarker for detecting early subclinical renal tubular stress [10], correlating with severity of ischemic tubular injury [11]. Beyond diagnosis, some studies have evaluated urinary L-FABP for risk stratification and prediction of adverse renal outcomes [12,13]. Nevertheless, its role in exercise physiology remains under investigation.

Although strenuous exercise is known to induce transient elevations in urinary acute kidney injury (AKI) biomarkers [14], the precise dose–response relationship between exercise intensity and renal proximal tubular workload remains insufficiently defined. Previous investigations evaluating exertion-induced tubular stress have focused predominantly on extreme endurance events (e.g., marathon races) [15,16]. Therefore, whether acute physical activity, which was quantified continuously via metabolic equivalents of task (METs), exhibits a linear association with proximal tubular stress, or whether a distinct physiological threshold exists beyond which exercise-induced subclinical tubular stress accelerates nonlinearly, remains unknown.

To address this gap, we conducted a cohort study in young healthy men undergoing continuous real-time accelerometer monitoring during exercise. The primary objectives of this study were to characterize the dose–response relationship between exercise intensity and post-exercise urinary L-FABP concentrations and identify an exploratory intensity threshold for exertion-induced subclinical tubular stress.

## 2. Results

### 2.1. Clinical and Laboratory Characteristics of Participants

The final study cohort comprised 285 healthy young men who successfully completed the 20 min physical training (Figure 1). Based on measured physical activity concentrations during exercise, 176 participants (61.8%) were categorized into the moderate-intensity exercise group (3.0–5.9 METs) and 109 participants (38.2%) into the vigorous-intensity exercise group (≥6.0 METs).

Baseline demographic, clinical, and laboratory parameters of the participants stratified by intensity are summarized in Table 1. Overall, participants in the vigorous-intensity group were slightly younger (median, 22 vs. 23 years; *p* < 0.001) and had lower baseline serum creatinine levels (median, 88 vs. 92 µmol/L; *p* = 0.02). No statistically significant differences were observed between the two intensity groups regarding BMI, mean arterial pressure, respiratory rate, resting pulse oximetry, BUN, eGFR, or baseline urinary L-FABP concentrations (median, 0.90 vs. 0.85 µg/gCr).

Following the exercise protocol, participants exhibited significant physiological responses, including increased heart rate (median, 110 vs. 86 beats/min), elevated mean arterial pressure (97 vs. 90 mm Hg), and increased respiratory rate (20 vs. 14 breaths/min) (all *p* < 0.001). We also identified slight but statistically significant post-exercise increases in BUN (median, 6.39 vs. 6.25 mmol/L; *p* = 0.02) and serum creatinine (95 vs. 90 µmol/L; *p* < 0.001) (Table 2).

Post-exercise urinary L-FABP concentrations exhibited a marked elevation compared with baseline concentrations (median, 1.89 vs. 0.90 µg/gCr; *p* < 0.001). The median paired difference between pre- and post-exercise urinary L-FABP concentrations was 1.34 µg/gCr (95% CI, 1.15–1.56) (Figure 2).

### 2.2. Linear Association Between Exercise Intensity and Post-Exercise Urinary L-FABP Concentrations

Multivariable linear regression models evaluating the relationship between exercise intensity and post-exercise urinary L-FABP concentrations are shown in Table 3. When analyzed as a continuous variable, higher exercise intensity was independently associated with elevated post-exercise urinary L-FABP concentrations. In the unadjusted model (Model 1), each 1 MET increase in exercise intensity was associated with a 0.338 increase in post-exercise urinary L-FABP (β = 0.338; 95% CI, 0.280 to 0.395). This positive association remained statistically significant after adjusting for age and BMI in Model 2 (β = 0.348; 95% CI, 0.290 to 0.407), as well as after full adjustment for age, BMI, baseline eGFR, and baseline urinary L-FABP in Model 3 (β = 0.339; 95% CI, 0.284 to 0.394). Categorical analysis further confirmed that participants performing vigorous exercise experienced a significantly greater increase in post-exercise L-FABP concentrations compared with those in the moderate-intensity group in the fully adjusted model (β = 0.640; 95% CI, 0.499 to 0.782). Stratified subgroup analyses demonstrated that the linear association between exercise intensity and post-exercise urinary L-FABP was highly consistent across age, BMI, eGFR, and baseline L-FABP quartiles (all *p* for interaction > 0.10) (Figure 3).

### 2.3. Nonlinear Dose–Response Pattern and Threshold Estimation

Restricted cubic spline (RCS) modeling revealed a distinct nonlinear dose–response relationship between exercise intensity and post-exercise urinary L-FABP (*p* for overall association < 0.001; *p* for nonlinearity < 0.001) (Figure 4). The nonlinear RCS model provided a substantially superior fit compared with the linear model (AIC: 441.98 vs. 456.30, ΔAIC= −14.32; BIC: 474.86 vs. 481.87).

Subsequent piecewise linear regression analysis identified an empirical exercise intensity breakpoint at 6.76 METs (95% CI, 5.67–7.12) (Table 4). Below this threshold (<6.76 METs), exercise intensity showed no statistically significant association with post-exercise urinary L-FABP concentrations (β = 0.068; 95% CI, −0.077 to 0.213; *p* = 0.36), corresponding to a 7.0% increase per MET (95% CI, −7.4% to 23.7%). In contrast, above 6.76 METs, post-exercise urinary L-FABP concentrations escalated rapidly with increasing exercise intensity (β = 0.491; 95% CI, 0.383 to 0.599; *p* < 0.001), corresponding to a 63.4% increase per MET increase (95% CI, 46.7% to 82.0%). The difference in slopes before and after the breakpoint was statistically significant (Δβ = +0.423; 95% CI, 0.243 to 0.603; *p* < 0.001). The log-likelihood ratio test confirmed that the two-piecewise model provided a significantly better fit than the linear model (*p* < 0.001).

## 3. Discussion

In this cohort study of young men undergoing a 20 min exertion challenge, we investigated the continuous dose–response relationship between physical workload quantified via METs and post-exercise renal proximal tubular stress, as measured by urinary L-FABP concentrations. Our investigation yielded three principal findings: acute exertion induced a statistically significant, dose-dependent surge in urinary L-FABP relative to baseline, and modeling via RCS and piecewise linear regression revealed a distinct nonlinear trajectory with an exploratory threshold at 6.76 METs. Below 6.76 METs, urinary L-FABP excretion exhibited minimal change, whereas exceeding this threshold into the vigorous exertion domain triggered a remarkable escalation in biomarker shedding, marking an exploratory transition zone beyond which subclinical renal tubular workload increases.

The majority of prior studies regarding exertion-induced renal biomarker responses have focused on extreme endurance events, such as full marathons and ultramarathons. A study by Mansour et al. evaluated marathon runners and observed that 82% of participants developed an increase in creatinine level, accompanied by marked elevations in injury biomarker levels such as kidney injury molecule 1 and neutrophil gelatinase-associated lipocalin (NGAL) [17]. According to Poussel et al., ultramarathons did not cause severe renal impairment but transient elevated biomarkers such as cystatin C and NGAL [18]. However, these extreme endurance models present major methodological limitations. In marathons or ultramarathons, the isolated biological impact of exercise intensity is heavily confounded by prolonged cumulative exercise duration, severe thermal strain, systemic inflammatory cascades, fluid and electrolyte disturbances, hemolysis and skeletal muscle damage [19,20,21]. By employing a 20 min exertion protocol under the same conditions, our study could isolate acute exercise intensity as the primary independent exposure variable, proving that tubular stress could be triggered within a short time window purely as a function of physical workload.

In a clinical study on individuals without chronic kidney disease, Kosaki et al. (2020) [22] demonstrated that incremental short maximal exercise significantly increases urinary concentrations of albumin and L-FABP. The post-exercise percentage change in L-FABP correlated independently with age, baseline estimated glomerular filtration rate, and changes in urinary albumin (R^2^ = 0.451, *p* < 0.001) [22]. These findings suggested that short-duration maximal exertion can induce acute, mild adverse effects on tubular health, particularly in younger adults. Tominaga et al. similarly demonstrated elevated urinary L-FABP and oxidative stress markers following a 3000 m maximal time trial in male runners. The results indicated that acute, high-intensity endurance running acutely induces transient renal subclinical injury, highlighting the utility of urinary biomarkers for noninvasive monitoring of exercise-induced physiological stress [23]. Nevertheless, these previous investigations primarily focused on comparing pre- versus post-exercise biomarker concentrations and identifying associated clinical factors. They did not directly assess the continuous relationship between biomarker concentrations and physical workload or identify specific exertional thresholds [22,23]. Our study is among the first to model exercise intensity as a continuous variable (METs) and directly evaluate its dose–response relationship with urinary L-FABP. This approach enabled us to characterize an exploratory intensity breakpoint at 6.76 METs associated with exertional proximal tubular stress in this cohort, serving as a preliminary finding for exercise physiology.

During acute exercise, the cardiovascular system undergoes significant hemodynamic adaptations to meet the increased metabolic demands of skeletal muscle. Systemic blood flow is dynamically redistributed: local metabolic vasodilation, which is mediated by adenosine, potassium ions, and lactic acid, diverts up to 84% of total blood volume to active skeletal muscle, while sympathetic vasoconstriction restricts perfusion to splanchnic and renal vascular beds [24]. However, moderate-intensity exercise did not reduce renal blood flow and did not induce renal injury [25]. Renal injury biomarkers such as urinary kidney injury molecule-1 (uKIM-1) level exhibited no significant change immediately after exercise [25]. These findings align with our results, demonstrating that physical exertion below 6.76 METs does not induce a statistically or clinically significant increase in post-exercise urinary L-FABP concentrations (β_pre-breakpoint_ = 0.068; 95% CI, −0.077 to 0.213). During vigorous exertion, marked sympathetic activation drives profound renal afferent arteriolar vasoconstriction, reducing renal blood flow by 50–70% [26,27]. Once physical workload surpasses the estimated breakpoint, urinary L-FABP concentrations escalate exponentially.

Proximal tubular epithelial cells require substantial amounts of ATP to sustain solute reabsorption. Under physiological conditions, these cells predominantly rely on mitochondrial oxidative metabolism to generate large quantities of ATP, utilizing fatty acids as their primary fuel source. However, they have limited capacity for glycolysis. Consequently, during ischemia or hypoxia, mitochondrial function is impaired. This metabolic failure leads to the intracellular accumulation of free fatty acids [28]. L-FABP is predominantly expressed in proximal tubules. L-FABP facilitates the cellular uptake, transport, and metabolism of fatty acids [29]. Under conditions of renal tubular stress, L-FABP gene expression is rapidly upregulated, leading to its prompt excretion into the urine [30]. This proposed cellular mechanism may explain the acceleration in urinary L-FABP concentrations during vigorous exercise, as demonstrated in our findings.

This study has several limitations. First, our cohort was restricted to healthy young adult males, which limits the direct generalizability of our findings to females, older populations, or patients with underlying chronic kidney disease or reduced microvascular reserve. Second, we assessed urinary L-FABP only at a single 30 min post-exercise time point; serial urine sampling over a longer recovery window was not performed to trace the complete clearance kinetic curve back to baseline. Finally, while we identified a significant intensity threshold, these findings are exploratory and require further validation in independent cohorts before they can be utilized for precise clinical workload monitoring.

## 4. Materials and Methods

### 4.1. Study Design and Setting

We conducted an observational cohort study among participants undergoing routine physical training to investigate the dose–response association between exercise intensity and post-exercise urinary L-FABP concentrations. The study took place at 108 Military Central Hospital, Hanoi, Vietnam, in April 2022. We strictly adhered to the Strengthening the Reporting of Observational Studies in Epidemiology (STROBE) reporting guideline for observational studies. The investigation was conducted in accordance with the principles of the Declaration of Helsinki. The Institutional Review Board and Ethics Committee of 108 Military Central Hospital approved the study protocol (Approval No. 351, dated 21 January 2021). All participants provided written informed consent prior to enrollment.

### 4.2. Participants

We recruited young healthy men aged between 18 and 45 years participating in routine physical training. We excluded individuals with a known history of chronic kidney disease with baseline estimated glomerular filtration rate < 60 mL/min/1.73 m^2^, kidney stones, severe cardiovascular conditions, hypertension, diabetes mellitus, or active infectious diseases. Participants who consumed nephrotoxic drugs, nonsteroidal anti-inflammatory drugs (NSAIDs), alcohol, or high-protein supplements within 48 h prior to the exercise testing were also excluded.

### 4.3. Sample Size

We calculated the sample size using a model-based parametric Monte Carlo simulation (N = 2000 iterations per scenario) tailored for piecewise linear regression. We planned for five total predictors: exercise intensity (METs) as the primary independent variable and four baseline covariates (age, body mass index, baseline estimated glomerular filtration rate, and baseline urinary L-FABP). The simulation framework incorporated empirical baseline parameters derived from prior exercise study data (median baseline urinary L-FABP = 1.13 µg/g creatinine, IQR: 0.77–1.67; log-scale β_0_ = 0.122, β_1_ = 0.026, and residual error = 0.425 corresponding to an R^2^ = 0.451) [22]. Based on exercise-induced acute renal response physiology, we hypothesized a conservative minimum clinically important difference post-breakpoint slope change of β_2_ = 0.25 on the log scale at a hypothesized threshold of K = 6.0 METs (representing the physiological boundary between non-vigorous and vigorous exercise). Assuming a two-tailed α = 0.05, the simulation demonstrated that a sample size of N ≥ 175 participants achieves a statistical power exceeding 90% to successfully detect the nonlinear breakpoint effect.

### 4.4. Data Collection

We collected clinical characteristics, physiological metrics, and biological specimens at baseline (immediately before exercise) and after the exercise protocol. We recorded demographic parameters (age), physical measurements (body mass index [BMI], calculated as weight in kilograms divided by height in meters squared), and vital signs (heart rate, mean arterial pressure, respiratory rate, and pulse oxygen saturation [SpO2]) both before and after exercise. Venous blood samples were collected to measure blood urea nitrogen (BUN) and serum creatinine, and to calculate baseline eGFR using the Chronic Kidney Disease Epidemiology Collaboration (CKD-EPI) formula.

All participants performed their standard 20 min outdoor physical training session under the same environmental conditions. Participants engaged in their routine self-paced physical activities as part of their standard training. Physical activity intensity was continuously monitored throughout the 20 min session using a tri-axial MEMS accelerometer wrist device (ActiGraph wGT3X-BT, ActiGragh LLC, Pensacola, FL, USA; dynamic range ± 8 G) secured to the non-dominant wrist. Devices were initialized via ActiLife software (version 6.13.3, ActiGraph LLC) at a sampling frequency of 30 Hz. Individual biometric parameters (age, height, weight, sex) were pre-programmed into the software. Acceleration data were collected in 60 s epochs, and 3-axis vector magnitude counts (VM = $\sqrt{Axis1^{2}+Axis2^{2}+Axis3^{2}}$) were converted into participant-specific Metabolic Equivalents of Task using the validated Freedson Adult VM3 Combination algorithm (METs = 0.000863 × VM_cpm_ + 0.668879) with the “Worn on Wrist” configuration enabled in ActiLife v6.13.3. The mean MET value recorded over the 20 min exercise period was extracted for each participant to represent overall exercise intensity as a continuous variable.

At 10 min post-exercise, we remeasured all vital signs. We obtained midstream spot urine samples (5 mL per collection) from each participant at 2 distinct time points: immediately pre-exercise and 30 min post-exercise. Urine samples were centrifuged and analyzed. We quantified urinary L-FABP concentration using a commercial latex-enhanced turbidimetric immunoassay (LTIA) kit (Norudia L-FABP, Sekisui Medical Co., Ltd., Tokyo, Japan; Catalog No. 507823) on an automated clinical chemistry analyzer (Beckman Coulter PE Automated System, ISO 15189:2012 certified). Raw L-FABP was measured in ng/mL (µg/L) and spot urinary creatinine in g/L. To adjust for urinary dilution, we normalized all urinary L-FABP concentrations to urinary creatinine concentrations and we express the values as micrograms per gram of creatinine (µg/gCr).

### 4.5. Statistical Analysis

We analyzed all data using R software, version 4.4.1 (R Foundation for Statistical Computing). We assessed the normality of continuous variables using the Shapiro–Wilk test. Continuous variables are expressed as the mean (SD) for normally distributed data or median (IQR) for non-normally distributed data. Categorical variables are presented as numbers and percentages.

We compared baseline characteristics between moderate-intensity and vigorous-intensity exercise groups using the Mann–Whitney *U* test. For paired pre- and post-exercise comparisons within the overall cohort, we used the Wilcoxon signed-rank test for continuous non-normally distributed metrics (including urinary L-FABP).

Due to the right-skewed distribution of urinary L-FABP concentrations, we log-transformed the values (ln[L-FABP]) prior to parametric modeling to satisfy the assumptions of normality and homoscedasticity of residuals.

We constructed multivariable linear regression models to evaluate the association between exercise intensity and post-exercise urinary L-FABP concentrations. To ensure consistency, we performed stratified analyses by age (≤median vs. >median, years), BMI defined according to the World Health Organization criteria for Asian populations (<23.0 vs. ≥23.0 kg/m^2^, corresponding to normal weight vs. overweight/obese), eGFR (<90 vs. ≥90 mL/min/1.73 m^2^), and baseline urinary L-FABP quartiles (Q1–Q4). We tested for interactions by incorporating multiplicative interaction terms into the fully adjusted models.

To evaluate potential nonlinear dose–response relationships, we modeled exercise intensity using restricted cubic splines (RCSs) with 4 knots placed at the 5th, 35th, 65th, and 95th percentiles of the MET distribution. To confirm whether the nonlinear approach improved model fit compared with the linear model, we performed a likelihood ratio test and evaluated the Akaike Information Criterion (AIC) and Bayesian Information Criterion (BIC). When we identified significant nonlinearity (*p* for nonlinearity < 0.05), we applied piecewise linear regression to determine the intensity threshold and estimate the precise breakpoint using maximum likelihood methods. Additionally, a bootstrapping with 1000 resamples was employed to calculate the 95% CI for the estimated breakpoint. The goodness-of-fit between the two-piecewise linear model and the one-segment linear model was further compared using a log-likelihood ratio test. We estimated independent regression coefficients (β), percentage changes in log-transformed urinary L-FABP and 95% CIs for segments both below and above the breakpoint, as well as the difference in slopes.

All statistical tests were 2-tailed, and a *p* < 0.05 was considered statistically significant.

## 5. Conclusions

In this cohort study of young healthy men, exercise intensity demonstrated a nonlinear, threshold-dependent dose–response association with post-exercise urinary L-FABP concentrations. We identified an exploratory threshold at 6.76 METs, beyond which post-exercise urinary L-FABP concentrations increase more rapidly. These findings provide preliminary evidence for monitoring exertion-associated renal workload, though the identified threshold is exploratory and requires prospective validation in external populations.

## Figures and Tables

**Figure 1:**
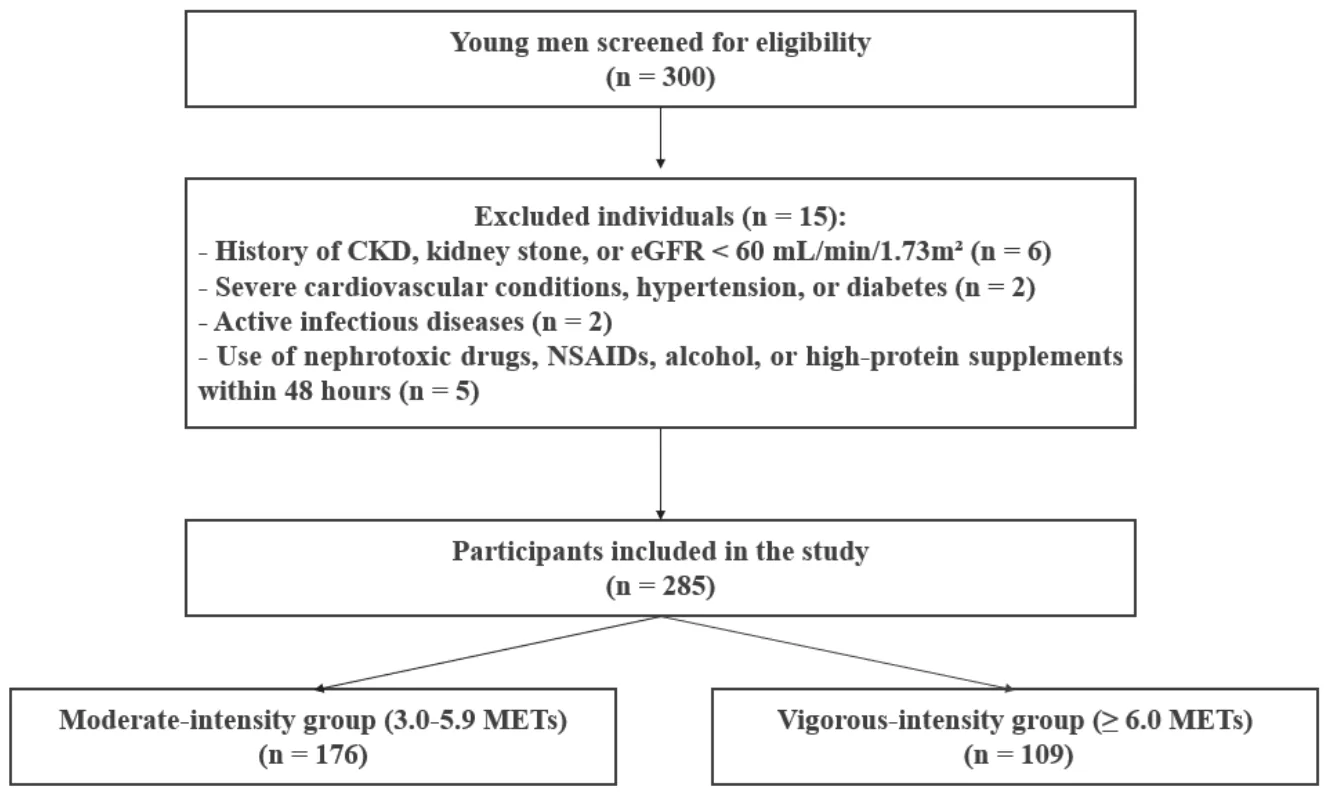
Flowchart of participants. A total of 300 young men were screened for study eligibility. Fifteen individuals were excluded based on predefined criteria, including a history of chronic kidney disease or baseline eGFR < 60 mL/min/1.73 m^2^ (*n* = 6); cardiovascular diseases, hypertension, or diabetes (*n* = 2); active infectious diseases (*n* = 2); and recent exposure to nephrotoxic drugs, NSAIDs, alcohol, or high-protein supplements within 48 h prior to testing (*n* = 5). Ultimately, 285 healthy men were included in the final analysis. Participants were categorized based on continuous physical activity monitoring into the moderate-intensity exercise group (3.0–5.9 METs, *n* = 176) and the vigorous-intensity exercise group (≥6.0 METs, *n* = 109). Abbreviations: CKD, chronic kidney disease; eGFR, estimated glomerular filtration rate; METs, metabolic equivalents of task; NSAIDs, nonsteroidal anti-inflammatory drugs.

**Figure 2:**
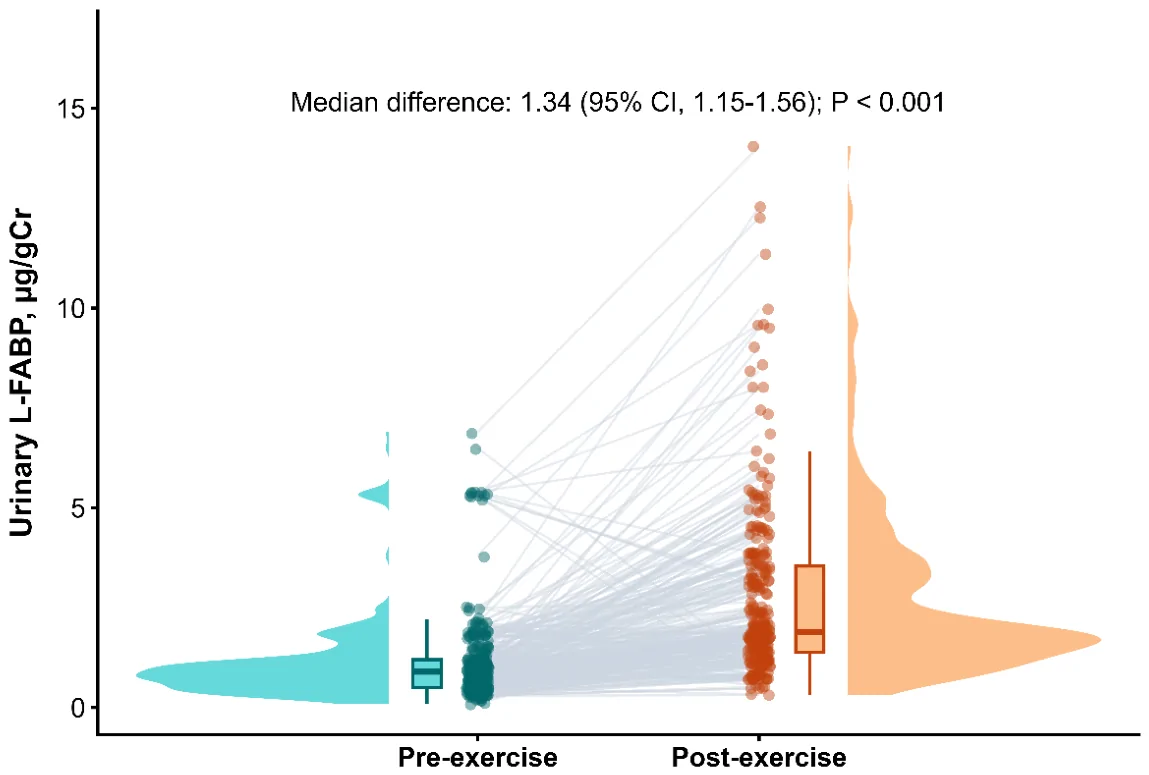
Paired comparison of urinary L-FABP concentrations pre- and post-exercise. L-FABP concentrations before (baseline) and after a 20 min exercise. Individual paired values are presented as combined boxplots, raincloud density distributions, and individual lines connecting pre- and post-exercise measurements for each participant (*n* = 285). In the boxplots, horizontal lines within the boxes represent the median values; lower and upper hinges correspond to the 25th and 75th percentiles; and whiskers extend to 1.5 × IQR. Pairwise comparison between pre- and post-exercise concentrations was analyzed using the two-tailed non-parametric Wilcoxon signed-rank test (*p* < 0.001). Abbreviations: gCr, gram of creatinine; L-FABP, liver-type fatty acid-binding protein.

**Figure 3:**
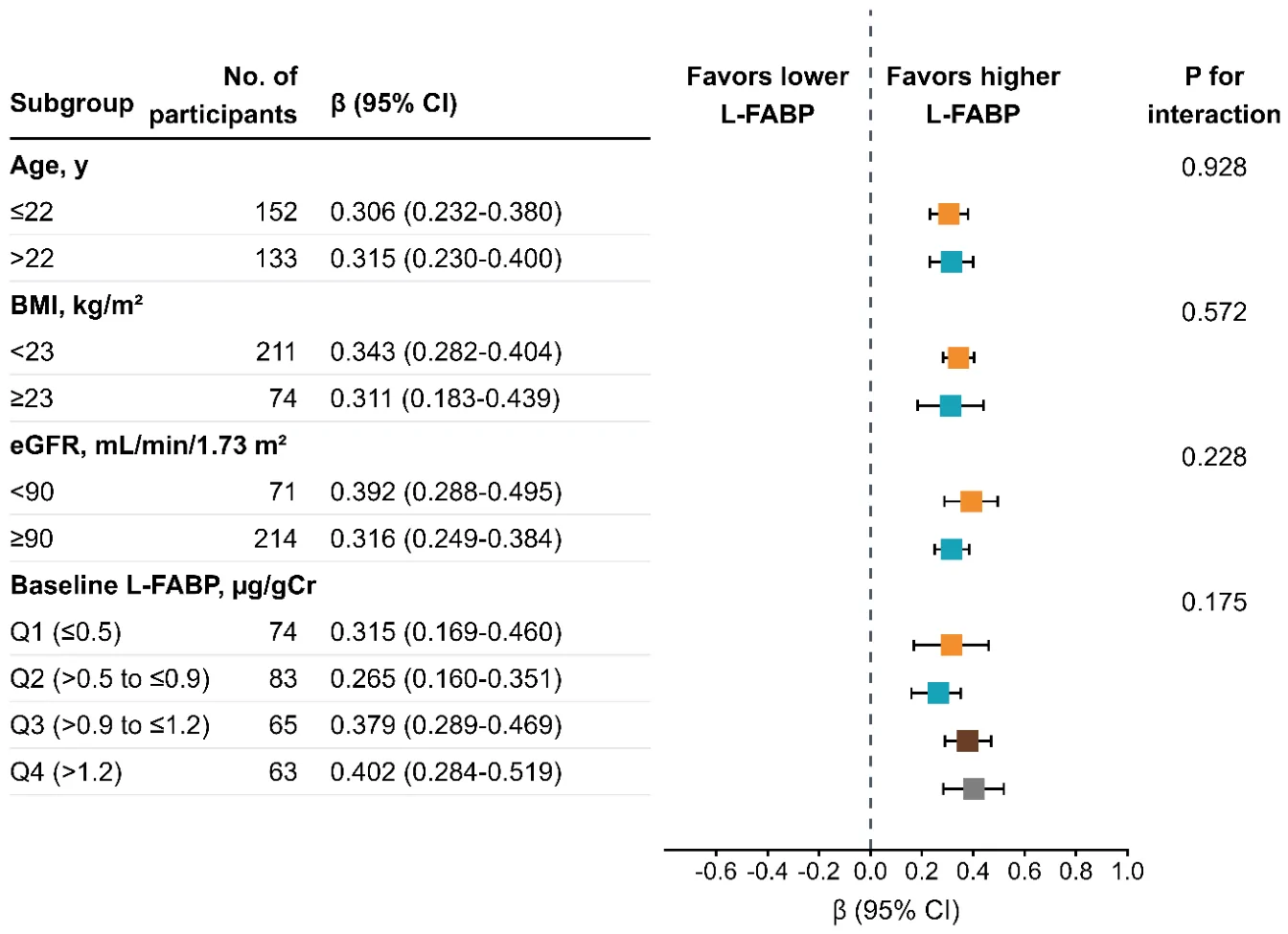
Subgroup analysis of the association between exercise intensity and post-exercise urinary L-FABP concentrations. Forest plot showing the adjusted regression coefficients (β) and 95% CIs for the association between exercise intensity (per 1-MET increase) and post-exercise natural log-transformed urinary L-FABP concentrations across clinical and demographic subgroups. Solid squares represent point estimates of regression coefficients (β); horizontal error bars indicate 95% CIs. All subgroup estimates were derived from multivariable linear regression models fully adjusted for age, BMI, baseline eGFR, and baseline log-transformed urinary L-FABP concentrations. *p*-values for interaction were calculated using multiplicative interaction terms in fully adjusted models to evaluate effect heterogeneity across subgroups. All statistical tests were 2-tailed, and *p* < 0.05 was considered statistically significant. Abbreviations: BMI, body mass index; CI, confidence interval; eGFR, estimated glomerular filtration rate; L-FABP, liver-type fatty acid-binding protein; Q, quartile.

**Figure 4:**
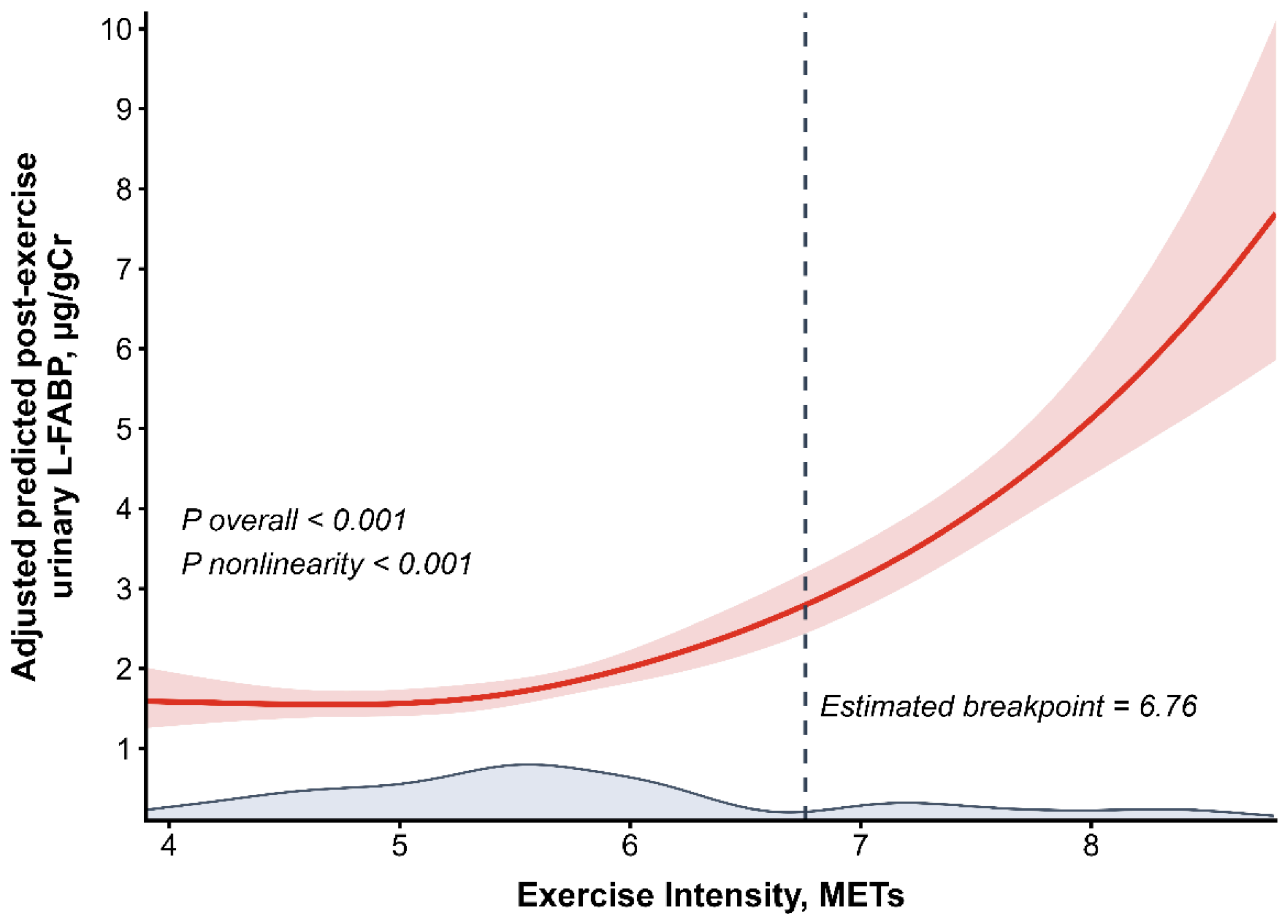
Dose–response relationship between exercise intensity and post-exercise urinary L-FABP using restricted cubic splines. Restricted cubic spline (RCS) regression models illustrating the dose–response association between exercise intensity (in METs) and post-exercise natural log-transformed urinary L-FABP concentrations. The solid red line represents the continuous fitted smooth curve modeled using 4 knots placed at the 5th, 35th, 65th, and 95th percentiles of the MET distribution; the shaded red area indicates the surrounding 95% confidence interval (CI). The dashed gray vertical line marks the identified exercise intensity threshold (breakpoint) at 6.76 METs, estimated via maximum likelihood segmented linear regression methods. The model was fully adjusted for age, body mass index, baseline eGFR, and baseline log-transformed urinary L-FABP concentrations. The *p*-value for overall association was <0.001, and the *p*-value for nonlinearity was <0.001. All statistical tests were 2-tailed. Abbreviations: CI, confidence interval; gCr, gram of creatinine; L-FABP, liver-type fatty acid-binding protein; METs, metabolic equivalents of task; RCS, restricted cubic spline.

**Table 1:** Baseline characteristics of participants.

Variables	Exercise Intensity		*p*-Value
	Moderate (3.0–5.9 METs)	Vigorous (≥6 METs)	
	*n* = 176	*n* = 109	
Age, years (median [IQR])	23 (21–26)	22 (21–23)	<0.001
BMI, kg/m^2^ (median [IQR])	21.58 (20.31–23.49)	21 (20.2–22.5)	0.108
Mean arterial pressure ^†^, mmHg (median [IQR])	90 (86–93.33)	90 (84–93.33)	0.757
Respiratory rate, breaths/min (median [IQR])	14 (14–17)	15 (14–17)	0.559
SpO_2_, % (median [IQR])	99 (97–99)	99 (96.5–99)	0.05
BUN, mmol/L (median [IQR])	6.31 (5.33–7.10)	6.11 (5.59–7.20)	0.757
Serum creatinine, µmol/L (median [IQR])	92 (85–100)	88 (83–97)	0.024
eGFR ^‡^, mL/min/1.73 m^2^, (median [IQR])	96 (90–105)	99 (88.5–106)	0.309
Baseline L-FABP, µg/gCr (median [IQR])	0.85 (0.53–1.20)	0.9 (0.5–1.2)	0.883

Continuous variables are presented as median (IQR) because they were non-normally distributed as assessed by the Shapiro–Wilk test. Comparisons between exercise intensity groups (moderate vs. vigorous) were performed using the non-parametric Mann–Whitney *U* test. All tests were 2-tailed, and *p* < 0.05 was considered statistically significant. Abbreviations: METs, metabolic equivalents of task; BMI, body mass index; SpO_2_, pulse oximetry saturation; BUN, blood urea nitrogen; eGFR, estimated glomerular filtration rate; L-FABP, liver-type fatty acid-binding protein; gCr, gram of creatinine. Mean arterial pressure ^†^ was calculated using the standard formula: MAP = (systolic blood pressure + 2 × diastolic blood pressure)/3. ^‡^ Calculated using the Chronic Kidney Disease Epidemiology Collaboration (CKD-EPI) creatinine equation.

**Table 2:** Comparison of parameters pre- and post-exercise.

Variables	Pre-Exercise	Post-Exercise	*p*-Value
Heart rate, beats/min (median [IQR])	86 (80–96)	110 (100–121)	<0.001
Mean arterial pressure, mmHg (median [IQR])	90 (86.67–95.33)	97 (93–102)	<0.001
Respiratory rate, breaths/min (median [IQR])	14 (14–17)	20 (18–24)	<0.001
SpO_2_, % (median [IQR])	99 (97–99)	98 (97–99)	0.005
BUN, mmol/L (median [IQR])	6.25 (5.35–7.16)	6.39 (5.61–7.16)	0.02
Serum creatinine, µmol/L (median [IQR])	90 (84–98)	95 (89–103)	<0.001
Urinary L-FABP, µg/gCr (median [IQR])	0.9 (0.5–1.2)	1.89 (1.39–3.54)	<0.001

Continuous variables are presented as median (IQR) because they were non-normally distributed as assessed by the Shapiro–Wilk test. Paired comparisons between pre-exercise baseline values and post-exercise measurements were analyzed using the two-tailed Wilcoxon signed-rank test. All tests were 2-tailed, and *p* < 0.05 was considered statistically significant. Abbreviations: SpO_2_, pulse oximetry saturation; BUN, blood urea nitrogen; L-FABP, liver-type fatty acid-binding protein; gCr, gram of creatinine.

**Table 3:** Linear regression analysis for the association between exercise intensity and post-exercise urinary L-FABP concentrations.

Exercise Intensity	Model 1 ^†^	Model 2 ^‡^	Model 3 ^§^
	β 95% CI	β 95% CI	β 95% CI
Continuous (per MET increase)	0.338 (0.280–0.395)	0.348 (0.290–0.407)	0.339 (0.284–0.394)
Categorical			
Moderate	1.00 (Reference)	1.00 (Reference)	1.00 (Reference)
Vigorous	0.630 (0.481–0.778)	0.642 (0.490–0.794)	0.640 (0.499–0.782)

Multivariable linear regression models were constructed with natural log-transformed post-exercise urinary L-FABP concentrations treated as the continuous dependent variable. Abbreviations: CI, confidence interval; BMI, body mass index; eGFR, estimated glomerular filtration rate; L-FABP, liver-type fatty acid-binding protein; METs, metabolic equivalents of task. ^†^ Model 1. Crude model, no covariate was adjusted. ^‡^ Model 2. Adjusted for age and BMI. ^§^ Model 3. Adjusted for age, BMI, baseline eGFR and baseline natural log-transformed urinary L-FABP concentrations.

**Table 4:** Threshold effect analysis of exercise intensity on post-exercise urinary L-FABP.

	β 95% CI	% Change (95% CI)	*p*-Value
Estimated breakpoint	6.76 (5.67–7.12)	NA	NA
Slope before breakpoint	0.068 (−0.077–0.213)	7.0 (−7.4–23.7)	0.358
Slope after breakpoint	0.491 (0.383–0.599)	63.4% (46.7–82.0)	<0.001
Difference in slopes	+0.423 (0.243–0.603)	52.7 (27.5–82.7)	<0.001

Piecewise linear regression was performed with natural log-transformed post-exercise urinary L-FABP concentrations as the continuous dependent variable. The optimal exercise intensity breakpoint was determined using maximum likelihood estimation. The piecewise model was fully adjusted for age, body mass index, baseline estimated glomerular filtration rate, and baseline natural log-transformed urinary L-FABP concentrations. All tests were 2-tailed, and *p* < 0.05 was considered statistically significant. Abbreviations: CI, confidence interval; NA, not applicable.

## Data Availability

The original contributions presented in this study are included in the article. Further inquiries can be directed to the corresponding authors.

## Abbreviations

AKI	Acute Kidney Injury
ATP	Adenosine Triphosphate
BMI	Body Mass Index
BUN	Blood Urea Nitrogen
CI	Confidence Interval
CKD	Chronic Kidney Disease
CKD-EPI	Chronic Kidney Disease Epidemiology Collaboration
eGFR	Estimated Glomerular Filtration Rate
KIM-1/uKIM-1	(Urinary) Kidney Injury Molecule-1
L-FABP	Liver-type Fatty Acid-Binding Protein
MET	Metabolic Equivalent of Task
NGAL	Neutrophil Gelatinase-Associated Lipocalin
NSAIDs	Nonsteroidal Anti-inflammatory Drugs
RCSs	Restricted Cubic Splines
SpO2	Pulse Oxygen Saturation
VM	Vector Magnitude
VM_cpm_	Vector Magnitude Counts Per Minute
